# Impact of mineral and bone disorder on healthcare resource use and associated costs in the European Fresenius medical care dialysis population: a retrospective cohort study

**DOI:** 10.1186/1471-2369-13-140

**Published:** 2012-10-29

**Authors:** Silvia Chiroli, Caroline Mattin, Vasily Belozeroff, Louise Perrault, Dominic Mitchell, Ioanna Gioni

**Affiliations:** 1 Amgen (Europe) GmbH, Dammstrasse 23, 6300, Zug, Switzerland; 2 Amgen Ltd., Cambridge, United Kingdom; 3 Amgen Inc., Thousand Oaks, CA, USA; 4 International Market Access Consulting, Zug, Switzerland; 5 International Market Access Consulting, Montreal, QC, Canada; 6 Amgen Ltd., Uxbridge, United Kingdom

## Abstract

**Background:**

Secondary hyperparathyroidism (SHPT) is associated with mortality in patients with chronic kidney disease (CKD), but the economic consequences of SHPT have not been adequately studied in the European population. We assessed the relationship between SHPT parameters (intact parathyroid hormone [iPTH], calcium, and phosphate) and hospitalisations, medication use, and associated costs among CKD patients in Europe.

**Methods:**

The analysis of this retrospective cohort study used records of randomly selected patients who underwent haemodialysis between January 1, 2005 and December 31, 2006 at participating European Fresenius Medical Care facilities in 10 countries. Patients had ≥ 1 iPTH value recorded, and ≥ 1 month of follow-up after a 3-month baseline period during which SHPT parameters were assessed. Time at risk was post-baseline until death, successful renal transplantation, loss to follow-up, or the end of follow-up. Outcomes included cost per patient-month, rates of hospitalisations (cardiovascular disease [CVD], fractures, and parathyroidectomy [PTX]), and use of SHPT-, diabetes-, and CVD-related medications. National costs were applied to hospitalisations and medication use. Generalised linear models compared costs across strata of iPTH, total calcium, and phosphate, adjusting for baseline covariates.

**Results:**

There were 6369 patients included in the analysis. Mean ± SD person-time at risk was 13.1 ± 6.4 months. Patients with iPTH > 600 pg/mL had a higher hospitalisation rate than those with lower iPTH. Hospitalisation rates varied little across calcium and phosphate levels. SHPT-related medication use varied with iPTH, calcium, and phosphate. After adjusting for demographic and clinical variables, patients with baseline iPTH > 600 pg/mL had 41% (95% CI: 25%, 59%) higher monthly total healthcare costs compared with those with iPTH in the K/DOQI target range (150–300 pg/mL). Patients with baseline phosphate and total calcium levels above target ranges (1.13–1.78 mmol/L and 2.10–2.37 mmol/L, respectively) had 38% (95% CI: 27%, 50%) and 8% (95% CI: 0%, 17%) higher adjusted monthly costs, respectively. Adjusted costs were 25% (95% CI: 18%, 32%) lower among patients with baseline phosphate levels below the target range. Results were consistent in sensitivity analyses.

**Conclusions:**

These data suggest that elevated SHPT parameters increase the economic burden of CKD in Europe.

## Background

A significant proportion of patients with chronic kidney disease (CKD) progress to end-stage renal disease (ESRD), corresponding to CKD Stage 5, the National Kidney Foundation’s Kidney Disease Outcomes Quality Initiative (K/DOQI) classification for kidney failure [1]. Patients with ESRD generally require dialysis or transplantation [1], and those living with the disease experience increased morbidity, including high rates of hospitalisation for infection, cardiovascular interventions, and inpatient vascular access procedures [2].

Secondary hyperparathyroidism (SHPT) is one of the common sequelae of ESRD that contributes to excess morbidity and mortality. The key manifestation of SHPT is an increase in parathyroid hormone (PTH) levels due to decreased renal function and inhibition of mineral metabolism [3]. Recent Kidney Disease: Improving Global Outcomes (KDIGO) guidelines suggest maintaining intact plasma PTH (iPTH) levels for dialysed patients in CKD Stage 5 within approximately two to nine times the upper normal limit for the assay being used [4]. Prior to 2009, however, dialysis centres followed the K/DOQI guidelines that advocated a target iPTH range of 150–300 pg/mL for patients in CKD stage 5 [5]. In Europe an estimated 27% of patients with ESRD have iPTH higher than the K/DOQI target range [6].

Elevated iPTH levels in dialysis patients are associated with increased risk of fracture [7,8], extraskeletal calcification [9,10], renal osteodystrophy [11], and cardiovascular disease (CVD) [12-14].

SHPT and abnormal calcium and phosphate metabolism pose a growing challenge to healthcare systems, accounting for a major proportion of the healthcare resource utilisation and costs associated with treatment of ESRD and earlier stages of CKD [15]. Abnormal serum levels of PTH, phosphate, and calcium were found to be significantly associated with hospitalisation rates in dialysis patients in the US [16]. In a retrospective analysis, Schumock et al. [17] examined healthcare utilisation and costs among diabetic pre-ESRD CKD patients with and without SHPT, identified on the basis of diagnostic codes. Utilisation and costs (in 2004 US$) of prescription drugs, outpatient services, and hospitalisations were all significantly higher in patients with SHPT than in those without SHPT, even after multivariate adjustment for demographic and clinical covariates. Total healthcare costs were 320% higher in diabetic CKD patients with SHPT compared with those without SHPT [17]. Khan et al. [18] estimated the cost contribution of elevated iPTH levels in CKD patients in the US with congestive heart failure (CHF), all in stages prior to ESRD at baseline. Patients with iPTH ≥ 65 pg/mL had US$205 higher mean monthly CHF-related hospitalisation costs (in 2005 US$) in the period 1 to 3 months preceding dialysis compared with patients with iPTH < 65 pg/mL (below the upper bound of the K/DOQI target range for patients with Stage 3 CKD: 35–70 pg/mL [5]).

Given the differences in clinical practice patterns and patient populations that exist between Europe and the US, it is unknown whether a relationship exists between SHPT markers and healthcare resource use and costs in European patients. To better understand the economic burden of SHPT in ESRD patients in Europe, we assessed the relationship between levels of SHPT parameters and SHPT-related hospitalisation, medication use, and associated costs in a cohort of patients in both Western and Eastern Europe from the European Fresenius Medical Care (EU-FME) haemodialysis (HD) network.

## Methods

### Source population

The analysis of data from a retrospective cohort study used records from the European Clinical Database (EuCliD), a registry of medical data from HD patients at EU-FME dialysis centres, methodological details of which have been previously described [19]. Briefly, EuCliD contains detailed patient-level information on medical and drug history for all HD patients registered with the EU-FME, including records of key biochemical measurements and medications, and data on patient diagnoses, hospitalisations, and death codes based on the World Health Organization’s International Classification of Diseases, 10^th^ Revision (ICD-10) coding scheme. The present analysis comprised EuCliD data for randomly selected patients who underwent HD between January 1, 2005 and December 31, 2006 at participating EU-FME dialysis facilities in 10 countries: Czech Republic, France, Hungary, Italy, Poland, Portugal, Slovak Republic, Slovenia, Spain, and Turkey. Selection criteria and characteristics of this source population have been reported elsewhere [20]. Patients attending centres where the majority of data on key dialysis parameters were missing were excluded, as were patients from the UK because information on medication use was not available for UK centres [20]. Patient data were anonymised, and informed consent was obtained from all patients by EU-FME [20].

The cohort study did not include any human or animal experimentation for which ethical approval was required. All ethical and regulatory obligations concerning the use of patient data were met at each participating European Fresenius Medical Care study site. Because our analysis used existing data, there were no risks posed to human life. All patient information had been anonymised by Fresenius Medical Care prior to transfer of data for analysis. Permission was received from Fresenius Medical Care to use the database for this analysis. Fresenius Medical Care data were provided through a data license agreement which covered observational research use.

### Patient selection

To be eligible for the present analysis, patients were required to have at least one iPTH value recorded, and at least 1 month of follow-up after a 3-month baseline period during which SHPT parameters (iPTH, total calcium, and phosphate) were assessed. The baseline period was defined as the 3-month period starting on the date of the first recorded iPTH measurement. The SHPT parameters were defined as the mean value of the above laboratory measures taken during the 3-month baseline period. Since these parameters fluctuate over time [21], use of mean values during the 3-month period rather than at a single assessment helped to minimise potential exposure misclassification. Time at risk was defined as the time after the 3-month baseline period until death, successful renal transplantation, loss to follow-up, or the end of follow-up (December 31, 2006).

### Outcomes

Healthcare resource utilisation outcomes for this study included SHPT-related hospitalisations and medications. Hospitalisations potentially related to SHPT were defined as those associated with CVD, fracture, and parathyroidectomy (PTX). EuCliD records for the selected patients were used to calculate both the rate per 100 patient-years and the 12-month cumulative incidence of relevant hospitalisations, identified on the basis of ICD-10 codes as listed in Additional file 1: Table S1. CVD-, fracture-, and PTX-related hospitalisations were assessed separately and also combined as all–SHPT-related hospitalisations.

SHPT-related medications identified in the database included phosphate binders, oral vitamin D sterols, and Mimpara® (cinacalcet). Drugs for treating diabetes and CVD were also assessed separately. Included drugs are listed in Additional file 2: Table S2. Medication outcomes were number of days of treatment per patient-year and percentage of patients prescribed these medications.

Economic outcomes were the cost per month of CVD-, fracture-, and PTX-related hospitalisations, as well as the monthly cost of medications associated with treatment of SHPT, diabetes, and CVD. Diabetes and CVD medications were included because both comorbidities are associated with elevated SHPT parameters [13,22] and with morbidity and mortality in ESRD patients on HD [16,23]. The cost of each episode of hospitalisation related to CVD, fracture, or PTX was calculated by mapping the relevant ICD-10 code to country-specific diagnosis-related groups (DRGs) and their associated costs. French DRGs were used for costing for Turkey, where national DRG costs were not yet available. French DRGs were chosen over other nations’ for this purpose because they offered the combination of being available online, well documented, and weighted by different diagnoses. Costs of each episode of medication use were computed by mapping the medication and prescribed dose to an associated cost using national price lists for each country. Cost analyses included costs for both SHPT-related and non–SHPT-related (i.e., diabetes- and CVD-related) drug use. Hospitalisation and medication price lists were obtained for 2006 wherever available, with missing prices retrieved for other years and adjusted for inflation to 2006 Euros using the healthcare component of the Consumer Price Index.

Outcomes were stratified by baseline iPTH, total calcium, and phosphate. Values for each of these SHPT parameters were divided into clinically relevant categories, using the K/DOQI target range for each parameter for CKD Stage 5 as the reference category: 150–300 pg/mL for iPTH, 2.10–2.37 mmol/L for total calcium, and 1.13–1.78 mmol/L for phosphate [5]. Target ranges from the 2003 K/DOQI guidelines [5] were used rather than those from the 2009 KDIGO guidelines [4] because the former were followed during the study period (2006).

All countries were included in the analyses of healthcare resource utilisation. The cost analyses comprised patients only from the following five countries with ≥ 1000 patients enrolled in EU-FME/EuCliD: Hungary, Italy, Portugal, Spain, and Turkey.

### Statistical analysis

Hospitalisation rates were calculated by summing the number of relevant hospitalisation episodes across all patients and dividing by the person-time at risk for all patients. The 95% confidence interval was calculated assuming a Poisson distribution.

One-year cumulative incidence of hospitalisation (all-cause, as well as related to CVD, fractures, or PTX) was obtained with the Kaplan–Meier method and 95% confidence intervals using the arcsine-square root transformation. Data for patients not hospitalised were censored at the last known follow-up date or the end of follow-up (December 31, 2006).

The number of days of treatment per patient-year with phosphate binders, oral vitamin D sterols, and cinacalcet was calculated by summing the number of days when these medications were received during the time at risk and dividing by the person-time at risk.

The total cost per month was defined for each patient as the sum of costs for hospitalisations (CVD-, fracture-, and PTX-related) and medications (for SHPT, diabetes, and CVD) divided by the number of months at risk.

Univariate and multivariate analyses exploring the association of baseline SHPT parameters with cost per month were performed using generalised linear models (GLM) with a gamma probability distribution and a log link function. To account for patients with zero costs in the analyses, €1 was added to the total cost per month.

The univariate analysis for each SHPT parameter was adjusted for the duration of follow-up. The multivariate analysis included all SHPT parameters and duration of follow-up, and was also adjusted for the following potentially confounding factors: age, sex, CKD aetiology, history of CVD, diabetes and cancer, dialysis vintage, C-reactive protein, albumin, haemoglobin, ferritin, total cholesterol, and blood leukocyte count.

Results are presented for observed data, with no imputation of missing values. All statistical analyses were performed using SAS® (version 9.1, Cary, NC).

### Sensitivity analyses

To assess heterogeneity in duration of follow-up times between patients, a sensitivity analysis was conducted to compare results for patients with fixed periods of follow-up, defined as starting after the baseline period. The multivariate GLM in the main analysis was repeated for patients with a fixed follow-up time of 3 months, and again for patients with a fixed follow-up time of 6 months. These two sensitivity analyses included only patients with at least 3 and 6 months of follow-up, respectively, and in the analyses their follow-up time was truncated to these fixed periods.

In addition to the main analysis based on a one-part model, a two-part multivariate model was fitted in order to assess if the choice of the €1 additive factor in the cost per month affected the results. A two-part model is a mixture model in which the first part models the probability of a patient having a cost and the second part estimates the cost given a patient has a cost. The overall cost is the product of the first-part estimated probability and the second-part predicted conditional cost [24].

Lastly, to account for the presence of censored cost data, the weighted linear regression model proposed by Lin [25] was fitted. The study period was partitioned into several time intervals in order to include complete interval cost information where available. This approach aims to recreate the pseudo-population that would have been observed had no censoring occurred.

## Results

### Patient disposition and characteristics

**Figure 1:**
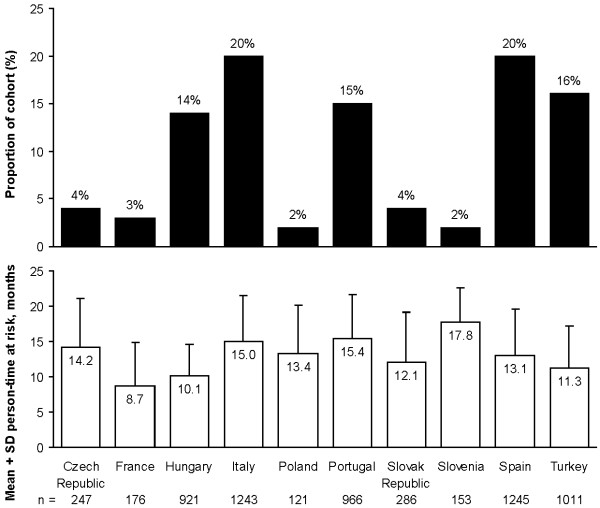
Patient geographic distribution.

Of the 6369 patients included from the 10 countries (see Figure 1), 1496 (23%) discontinued before the end of the follow-up period: 857 (13%) died, 373 (6%) were lost to follow-up, and 266 (4%) underwent successful renal transplant. Mean ± SD person-time at risk for the study cohort was 13.1 ± 6.4 months (range 1–21 months).

The mean ± SD age of the cohort was 63.0 ± 14.7 years, and more than half (57%) were male (see Table 1). Approximately three quarters of patients had a history of CVD, and a quarter had a history of diabetes. Increased iPTH values during the baseline period were observed in younger patients, in those with a longer dialysis vintage, and in lower proportions of male and diabetic patients. Patients who completed follow-up had similar demographics and baseline characteristics to those who discontinued prior to the end of follow-up, though a higher percentage of non-completers than completers had a history of diabetes (30% vs. 23%, respectively) and CVD (83% vs. 76%, respectively) (see Additional file 3: Table S3). Non-completers also had higher mean C-reactive protein levels compared with completers (14.43 vs. 11.77 mg/L, respectively).

**Table 1:** Patient characteristics at the beginning of the study by baseline iPTH level, all 10 countries

	Baseline iPTH*, pg/mL						**Total**
	**< 75**	**≥ 75 – < 150**	**≥ 150 – ≤ 300**	**> 300 – ≤ 600**	**> 600**	**> 800**	
N patients	1217	1365	1690	1343	754	472	6369
Males, n (%)	641 (53)	809 (59)	1019 (60)	767 (57)	394 (52)	233 (49)	3630 (57)
Age, years	63.6 (14.7)	64.7 (14.1)	64.2 (14.2)	62.0 (14.8)	58.2 (15.7)	57.5 (15.6)	63.0 (14.7)
Dialysis vintage^†^, months	43.9 (52.7)	37.8 (47.2)	40.9 (53.0)	49.0 (62.2)	66.5 (64.8)	70.6 (64.9)	45.5 (56.0)
History, n (%)							
Diabetes	352 (29)	404 (30)	447 (26)	276 (21)	102 (14)	56 (12)	1581 (25)
CVD	978 (80)	1075 (79)	1302 (77)	1020 (76)	589 (78)	368 (78)	4964 (78)
CKD aetiology, n (%)							
Hypertension/vascular	167 (14)	189 (14)	249 (15)	189 (14)	92 (12)	64 (14)	886 (14)
Glomerulonephritis	196 (16)	234 (17)	263 (16)	229 (17)	146 (19)	95 (20)	1068 (17)
Diabetes	208 (17)	208 (15)	268 (16)	153 (11)	54 (7)	27 (6)	891 (14)
Tubulo-interstitial	178 (15)	187 (14)	220 (13)	171 (13)	120 (16)	80 (17)	876 (14)
Polycystic kidney disease	60 (5)	73 (5)	100 (6)	83 (6)	67 (9)	43 (9)	383 (6)
Miscellaneous	37 (3)	40 (3)	78 (5)	61 (5)	34 (5)	20 (4)	250 (4)
Unknown	257 (21)	309 (23)	392 (23)	348 (26)	190 (25)	116 (25)	1496 (23)
Missing	114 (9)	125 (9)	120 (7)	109 (8)	51 (7)	27 (6)	519 (8)

CKD, chronic kidney disease; CVD, cardiovascular disease; iPTH, intact parathyroid hormone.

*Mean iPTH during 3-month baseline period.

^†^Calculated from initiation of dialysis to the start of the 3-month baseline period.

Values are mean (SD) unless specified otherwise.

### Healthcare resource utilisation

**Figure 2:**
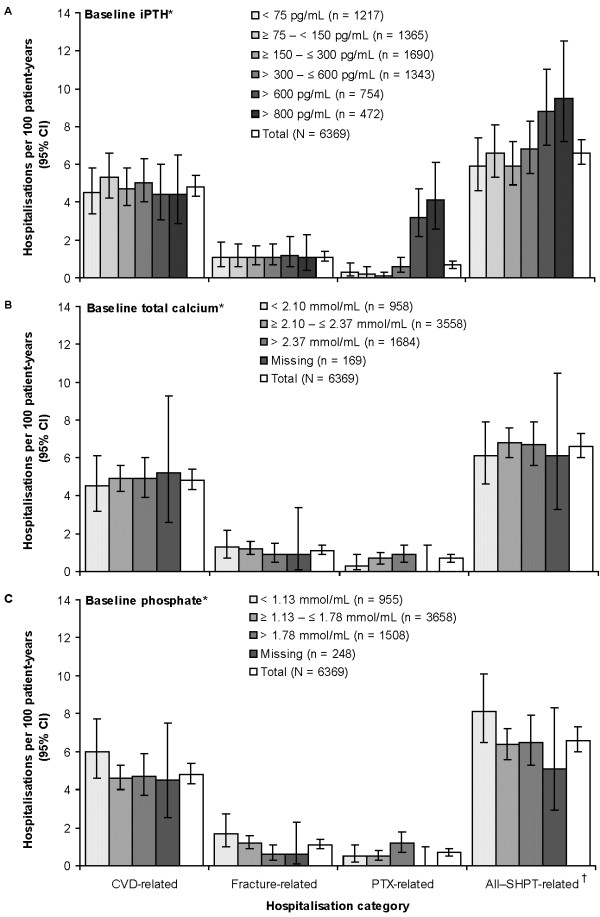
**Hospitalisations by baseline SHPT parameters, all 10 countries.** CVD, cardiovascular disease; iPTH, intact parathyroid hormone; PTX, parathyroidectomy; SHPT, secondary hyperparathyroidism. *Mean during 3-month baseline period. ^†^CVD-, fracture- or PTX-related.

Overall, the all–SHPT-related hospitalisation rate was 6.6 (95% CI: 6.0, 7.3) per 100 patient-years. Patients with baseline iPTH > 600 pg/mL had higher all–SHPT- and PTX-related hospitalisation rates than those with lower baseline iPTH (see Figure 2A). However, there was no obvious trend for CVD- and fracture-related hospitalisation rates across baseline iPTH levels. All–SHPT-related hospitalisation rates varied little across baseline total calcium levels (see Figure 2B). Similarly, there was no clear trend for all–SHPT-related hospitalisation rates to vary across baseline phosphate levels, although fracture-related hospitalisations decreased, and PTX-related hospitalisations increased with higher baseline phosphate (see Figure 2C). Findings were similar for 1-year cumulative incidence of hospitalisation (data not shown).

**Figure 3:**
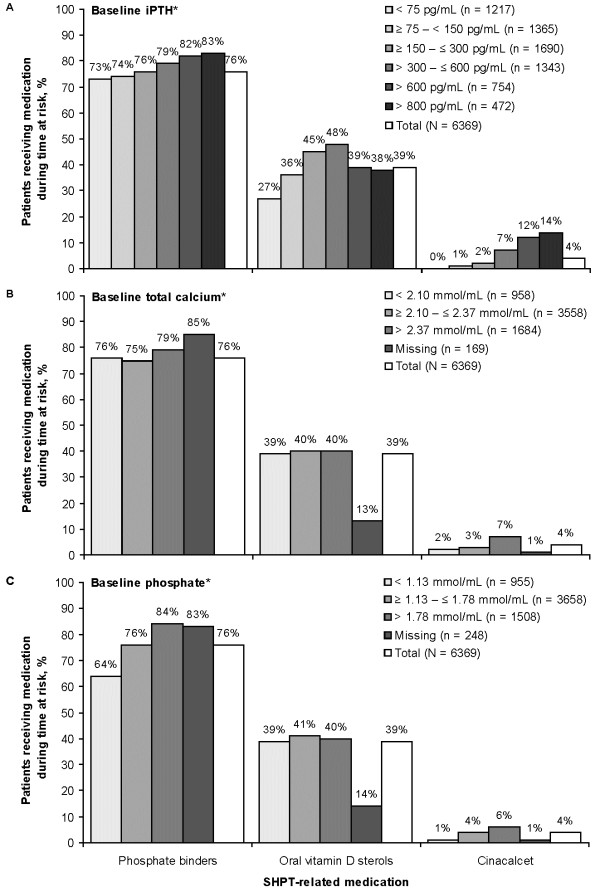
**Use of SHPT-related medications by baseline SHPT parameters, all 10 countries.** iPTH, intact parathyroid hormone. *Mean during 3-month baseline period.

**Figure 4:**
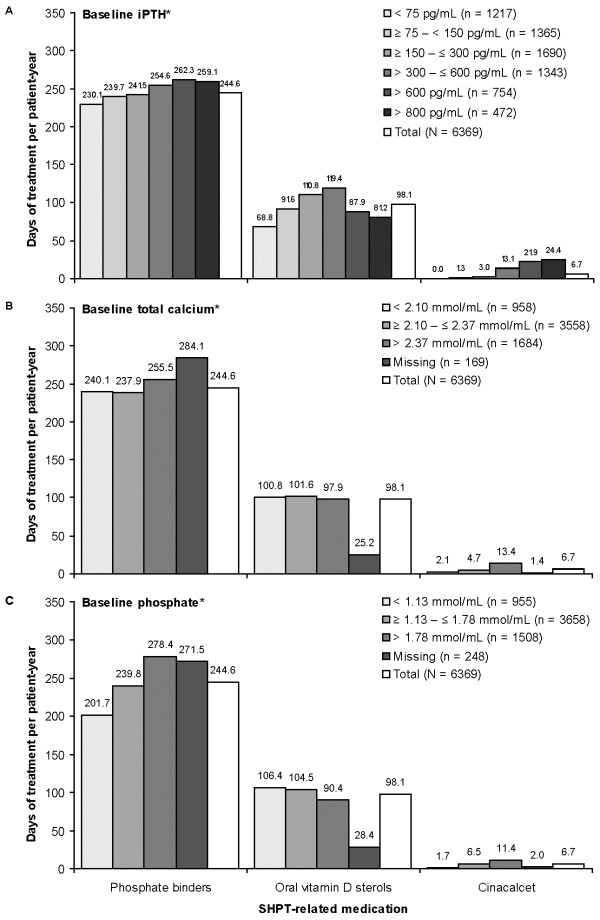
**Days of treatment with SHPT-related medications by baseline SHPT parameters, all 10 countries.** iPTH, intact parathyroid hormone. *Mean during 3-month baseline period.

During follow-up 76% of patients received phosphate binders, 39% received oral vitamin D sterols, and 4% received cinacalcet (see Figure 3). The percentage of patients using SHPT-related medications and the number of days of treatment per patient-year were both higher at elevated baseline iPTH levels for phosphate binders and cinacalcet, and were highest at intermediate baseline iPTH levels for oral vitamin D sterols (see Figure 3A and Figure 4A). The percentage of patients using medication and the number of days of treatment per patient-year were higher at elevated baseline total calcium levels for cinacalcet but not for phosphate binders or oral vitamin D sterols (see Figure 3B and Figure 4B). The percentage of patients using medication and the number of days of treatment per patient-year were higher at elevated baseline phosphate levels for cinacalcet and phosphate binders but not for oral vitamin D sterols (see Figure 3C and Figure 4C).

### Healthcare costs

The cost of healthcare resource utilisation (comprising the costs of CVD-, fracture-, and PTX-related hospitalisations and SHPT-, diabetes-, and CVD-related medications) increased with rising baseline iPTH (see Table 2). Mean monthly total healthcare costs were approximately €37 higher for patients with baseline iPTH > 600 pg/mL and €41 higher for those with baseline iPTH > 800 pg/mL compared with patients whose iPTH levels were within the K/DOQI target range at baseline (mean [interquartile range] €109.97 [14.88, 131.21], €114.12 [13.37, 132.83], and €73.47 [9.18, 79.33], respectively). Mean monthly medication costs increased with higher baseline iPTH, whereas there was no clear relationship between mean monthly hospitalisation costs and iPTH.

**Table 2:** Total healthcare cost per month by baseline iPTH, Hungary, Italy, Portugal, Spain, and Turkey

	Baseline iPTH*, pg/mL						**Total**
	**< 75**	**≥ 75 – < 150**	**≥ 150 – ≤ 300**	**> 300 – ≤ 600**	**> 600**	**> 800**	
N patients	1054	1145	1394	1140	653	405	5386
Total cost per month (including patients with zero costs), 2006 €							
Mean	60.52	67.01	73.47	82.46	109.97	114.12	75.89
SD	196.55	300.32	264.06	156.54	168.81	192.30	231.52
Median	16.15	22.34	26.66	41.68	57.69	56.57	28.61
Q1, Q3	5.01, 52.09	7.85, 69.08	9.18, 79.33	12.05, 101.16	14.88, 131.21	13.37, 132.83	8.55, 83.69
Min, Max	0.00, 3895.54	0.00, 9533.54	0.00, 7518.35	0.00, 3790.69	0.00, 2031.16	0.00, 2031.16	0.00, 9533.54
Patients with total cost per month > €0, n (%)	937 (89)	1052 (92)	1284 (92)	1060 (93)	605 (93)	372 (92)	4938 (92)
Cost of hospitalisations per month (including patients with zero costs), 2006 €							
Mean	23.40	23.21	21.50	17.87	25.51	29.49	21.95
SD	188.45	295.38	254.07	137.19	129.88	152.03	219.56
Median	0.00	0.00	0.00	0.00	0.00	0.00	0.00
Q1, Q3	0.00, 0.00	0.00, 0.00	0.00, 0.00	0.00, 0.00	0.00, 0.00	0.00, 0.00	0.00, 0.00
Min, Max	0.00, 3879.20	0.00, 9533.54	0.00, 7501.80	0.00, 3790.69	0.00, 1879.98	0.00, 1879.98	0.00, 9533.54
Patients with hospitalisation cost per month > €0, n (%)	40 (4)	50 (4)	51 (4)	63 (6)	50 (8)	34 (8)	254 (5)
Cost of medications per month (including patients with zero costs), 2006 €							
Mean	37.12	43.81	51.97	64.59	84.46	84.63	53.94
SD	54.32	56.55	70.41	76.68	103.14	107.58	72.70
Median	14.98	21.01	24.88	39.44	52.30	49.35	26.02
Q1, Q3	4.71, 48.12	7.34, 60.07	8.74, 71.46	11.20, 92.55	13.01, 112.43	11.38, 111.02	8.04, 74.56
Min, Max	0.00, 445.09	0.00, 418.67	0.00, 709.97	0.00, 698.08	0.00, 676.07	0.00, 676.07	0.00, 709.97
Patients with medication cost per month > €0, n (%)	933 (89)	1049 (92)	1281 (92)	1054 (92)	603 (92)	372 (92)	4920 (91)

iPTH, intact parathyroid hormone.

*Mean iPTH during 3-month baseline period.

Patients with baseline total calcium above the K/DOQI target range of 2.10–2.37 mmol/L had approximately €14 higher mean monthly costs than patients with baseline total calcium levels within the target range (mean [interquartile range] €86.09 [9.88, 104.82] vs. €72.57 [8.32, 79.72], respectively) (see Table 3). Mean monthly medication costs increased with higher baseline total calcium, but mean monthly hospitalisation costs did not show a clear trend in relation to total calcium.

**Table 3:** Total healthcare cost per month by baseline total calcium, Hungary, Italy, Portugal, Spain, and Turkey

	Baseline total calcium*, mmol/mL				**Total**
	**< 2.10**	**≥ 2.10 – ≤ 2.37**	**> 2.37**	**Missing**	
N patients	833	2980	1405	168	5386
Total cost per month (including patients with zero costs), 2006 €					
Mean	70.52	72.57	86.09	75.96	75.89
SD	308.29	233.85	171.97	171.79	231.52
Median	23.02	25.83	37.39	36.51	28.61
Q1, Q3	7.34, 68.65	8.32, 79.72	9.88, 104.82	9.87, 78.81	8.55, 83.69
Min, Max	0.00, 7518.35	0.00, 9533.54	0.00, 3790.69	0.00, 1766.00	0.00, 9533.54
Patients with total cost per month > €0, n (%)	759 (91)	2742 (92)	1274 (91)	163 (97)	4938 (92)
Cost of hospitalisations per month (including patients with zero costs), 2006 €					
Mean	26.33	21.08	19.91	32.71	21.95
SD	303.23	221.58	150.74	171.24	219.56
Median	0.00	0.00	0.00	0.00	0.00
Q1, Q3	0.00, 0.00	0.00, 0.00	0.00, 0.00	0.00, 0.00	0.00, 0.00
Min, Max	0.00, 7501.80	0.00, 9533.54	0.00, 3790.69	0.00, 1766.00	0.00, 9533.54
Patients with hospitalisation cost per month > €0, n (%)	33 (4)	136 (5)	74 (5)	11 (7)	254 (5)
Cost of medications per month (including patients with zero costs), 2006 €					
Mean	44.19	51.49	66.18	43.25	53.94
SD	61.73	70.83	83.31	40.75	72.70
Median	20.08	23.49	35.89	32.38	26.02
Q1, Q3	6.32, 59.11	7.89, 70.28	9.26, 91.74	8.51, 64.91	8.04, 74.56
Min, Max	0.00, 549.78	0.00, 709.97	0.00, 589.91	0.00, 159.19	0.00, 709.97
Patients with medication cost per month > €0, n (%)	754 (91)	2737 (92)	1269 (90)	160 (95)	4920 (91)

*Mean total calcium during 3-month baseline period.

Mean (interquartile range) monthly total healthcare costs increased with increasing baseline phosphate levels, from €62.02 (4.48, 54.32) among patients with phosphate < 1.13 mmol/L, to €75.27 (8.51, 82.41) among those in the K/DOQI target range of 1.13–1.78 mmol/L, and €88.88 (14.49, 107.77) among those with phosphate > 1.78 mmol/L (see Table 4). Mean monthly medication costs increased with higher baseline phosphate, while mean monthly hospitalisation costs were not clearly associated with phosphate levels.

**Table 4:** Total healthcare cost per month by baseline phosphate, Hungary, Italy, Portugal, Spain, and Turkey

	Baseline phosphate*, mmol/mL				**Total**
	**< 1.13**	**≥ 1.13 – ≤ 1.78**	**> 1.78**	**Missing**	
N patients	827	3104	1208	247	5386
Total cost per month (including patients with zero costs), 2006 €					
Mean	62.02	75.27	88.88	66.60	75.89
SD	176.01	273.40	145.08	147.07	231.52
Median	15.24	26.66	46.00	28.57	28.61
Q1, Q3	4.48, 54.32	8.51, 82.41	14.49, 107.77	10.65, 72.60	8.55, 83.69
Min, Max	0.00, 3790.69	0.00, 9533.54	0.00, 2031.16	0.00, 1766.00	0.00, 9533.54
Patients with cost per month > €0, n (%)	732 (89)	2856 (92)	1116 (92)	234 (95)	4938 (92)
Cost of hospitalisations per month (including patients with zero costs), 2006 €					
Mean	24.81	22.53	17.87	25.05	21.95
SD	165.02	264.57	111.07	142.43	219.56
Median	0.00	0.00	0.00	0.00	0.00
Q1, Q3	0.00, 0.00	0.00, 0.00	0.00, 0.00	0.00, 0.00	0.00, 0.00
Min, Max	0.00, 3790.69	0.00, 9533.54	0.00, 1879.98	0.00, 1766.00	0.00, 9533.54
Patients with hospitalisation cost per month > €0, n (%)	50 (6)	130 (4)	60 (5)	14 (6)	254 (5)
Cost of medications per month (including patients with zero costs), 2006 €					
Mean	37.21	52.74	71.01	41.55	53.94
SD	60.95	69.83	87.04	44.81	72.70
Median	13.45	24.96	41.28	27.11	26.02
Q1, Q3	3.89, 45.32	8.12, 74.40	13.37, 95.17	10.57, 62.62	8.04, 74.56
Min, Max	0.00, 549.78	0.00, 709.97	0.00, 676.07	0.00, 365.41	0.00, 709.97
Patients with medication cost per month > €0, n (%)	727 (88)	2848 (92)	1112 (92)	233 (94)	4920 (91)

*Mean phosphate during 3-month baseline period.

Additional file 4: Table S4, Additional file 5: Table S5, and Additional file 6: Table S6 present cost results for the component hospitalisation and medication categories stratified by baseline iPTH, total calcium, and phosphate levels, respectively.

The observed increase in costs with increasing iPTH persisted after adjustment for follow-up duration in the univariate analysis, and also after adjustment for follow-up duration and several demographic and clinical variables in the multivariate analysis (see Table 5). On average, patients with baseline iPTH > 600 pg/mL had 41% (95% CI: 25%, 59%) higher healthcare costs per month compared to patients with baseline iPTH levels in the K/DOQI target range, all else being equal. While the relative cost of healthcare resource utilisation increased with baseline total calcium above the reference range (i.e., > 2.37 mmol/L), the magnitude of this cost increase was small: 8% (95% CI: 0%, 17%) in the multivariate analysis (see Table 5). Relative costs were similar among patients with baseline total calcium below and within the reference range. In contrast, there was a 38% (95% CI: 27%, 50%) increase and a 25% (95% CI: 18%, 32%) decrease in relative cost of healthcare resource utilisation for patients with baseline phosphate above and below the reference range, respectively (i.e., > 1.78 mmol/L and < 1.13 mmol/L) (see Table 5).

**Table 5:** Relative healthcare cost per month by baseline SHPT parameters, Hungary, Italy, Portugal, Spain, and Turkey

Baseline level*	**n**	**Relative cost coefficient (95% CI)**	
		**Univariate analysis**^**†**^	**Multivariate analysis**^**‡**^
iPTH, pg/mL			
< 75	1054	1.01 (0.92, 1.12)	0.98 (0.89, 1.09)
≥ 75 – < 150	1145	0.98 (0.89, 1.08)	1.00 (0.91, 1.10)
≥ 150 – ≤ 300 (reference)	1394	1.00	1.00
> 300 – ≤ 600	1140	1.11 (1.01, 1.22)	1.11 (1.01, 1.22)
> 600	653	1.53 (1.36, 1.71)	1.41 (1.25, 1.59)
Total calcium (mmol/L)			
< 2.10 mmol/L	833	1.06 (0.97, 1.17)	1.01 (0.92, 1.11)
≥ 2.10 – ≤ 2.37 mmol/L (reference)	2980	1.00	1.00
> 2.37 mmol/L	1405	1.14 (1.06, 1.24)	1.08 (1.00, 1.17)
Missing	168	1.26 (1.03, 1.54)	1.54 (1.18, 2.00)
Phosphate (mmol/L)			
< 1.13 mmol/L	827	0.75 (0.68, 0.82)	0.75 (0.68, 0.82)
≥ 1.13 – ≤ 1.78 mmol/L (reference)	3104	1.00	1.00
> 1.78 mmol/L	1208	1.39 (1.28, 1.51)	1.38 (1.27, 1.50)
Missing	247	0.94 (0.79, 1.12)	0.70 (0.55, 0.88)

iPTH, intact parathyroid hormone.

*Mean during 3-month baseline period.

^†^Adjusted for duration of follow-up.

^‡^Adjusted for iPTH, total calcium, phosphate, duration of follow-up, age, sex, chronic kidney disease aetiology, history of cardiovascular disease, diabetes and cancer, dialysis vintage, C-reactive protein, albumin, haemoglobin, ferritin, total cholesterol and blood leukocyte count.

**Figure 5:**
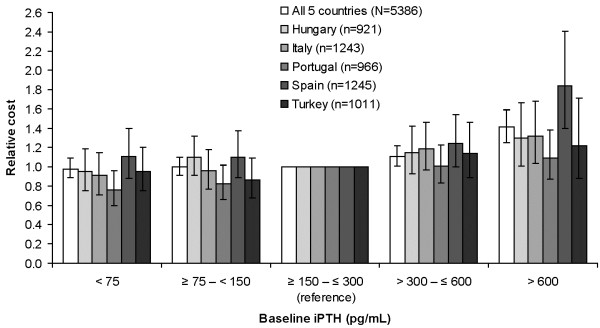
**Relative healthcare cost per month by baseline iPTH level.** * iPTH, intact parathyroid hormone. *Adjusted for total calcium, phosphate, duration of follow-up, and baseline factors. Error bars are 95% confidence intervals.

Associations between baseline iPTH level and relative healthcare costs for each of the five countries in the relative-cost analysis generally followed the trend for the five countries combined, although the trend appeared to be stronger in Spain and weaker in Portugal (see Figure 5). This may reflect different costs for hospitalisations and medications between countries. Portugal had more patients with missing values of baseline total calcium and phosphate compared to other countries for reasons we could not identify.

### Sensitivity analyses

In sensitivity analyses, the multivariate model was repeated for patients with fixed duration of follow-up time. Results trended in the same direction for patients with fixed follow-up times of 3 or 6 months (see Table 6). Relative costs in the two-part sensitivity analysis were consistent with relative costs in the main analysis, suggesting that addition of €1 to all monthly costs in the main analysis did not materially affect the results (see Table 6).

**Table 6:** Relative healthcare cost per month by baseline iPTH, total calcium, and phosphate in sensitivity analyses

Baseline level*	**Follow-up duration 3 months (N=5009)**		**Follow-up duration 6 months (N=4370)**		**Total healthcare cost > €0 (N=4938)**	
	**n**	**Relative Cost**^†^**(95% CI)**	**n**	**Relative Cost**^†^**(95% CI)**	**n**	**Relative Cost**^†‡^**(95% CI)**
iPTH, pg/mL						
< 75	965	0.90 (0.81, 1.00)	837	0.81 (0.72, 0.90)	937	1.00 (0.91, 1.10)
≥ 75 – < 150	1064	1.02 (0.92, 1.13)	928	1.00 (0.90, 1.11)	1052	0.99 (0.91, 1.09)
≥ 150 – ≤ 300 (reference)	1295	1.00	1124	1.00	1284	1.00
> 300 – ≤ 600	1068	1.14 (1.03, 1.27)	926	1.05 (0.95, 1.17)	1060	1.10 (1.00, 1.20)
> 600	617	1.50 (1.32, 1.70)	555	1.37 (1.20, 1.56)	605	1.40 (1.25, 1.57)
Total calcium (mmol/L)						
< 2.10 mmol/L	766	0.96 (0.87, 1.07)	631	0.95 (0.85, 1.06)	759	1.01 (0.92, 1.11)
≥ 2.10 – ≤ 2.37 mmol/L (reference)	2773	1.00	2397	1.00	2742	1.00
> 2.37 mmol/L	1311	1.03 (0.94, 1.12)	1195	1.00 (0.92, 1.10)	1274	1.10 (1.02, 1.19)
Missing	159	1.41 (1.02, 1.96)	147	1.14 (0.82, 1.58)	163	1.52 (1.18, 1.95)
Phosphate (mmol/L)						
< 1.13 mmol/L	764	0.77 (0.70, 0.86)	673	0.80 (0.71, 0.89)	732	0.78 (0.71, 0.85)
≥ 1.13 – ≤ 1.78 mmol/L (reference)	2889	1.00	2526	1.00	2856	1.00
> 1.78 mmol/L	1131	1.37 (1.25, 1.50)	960	1.44 (1.31, 1.58)	1116	1.38 (1.27, 1.50)
Missing	225	0.87 (0.64, 1.16)	211	0.85 (0.63, 1.15)	234	0.73 (0.59, 0.92)

iPTH, intact parathyroid hormone.

*Mean during 3-month baseline period.

^†^Based on pooled data for Hungary, Italy, Portugal, Spain, and Turkey, adjusted for iPTH, total calcium, phosphate, duration of follow-up, age, sex, chronic kidney disease aetiology, history of cardiovascular disease, diabetes and cancer, dialysis vintage, C-reactive protein, albumin, haemoglobin, ferritin, total cholesterol and blood leukocyte count.

^‡^Only the second-part results of the two-part model are presented.

A similar trend was observed in the sensitivity analysis using the Lin method [25] for all countries combined, with higher monthly mean costs occurring in patients with elevated baseline iPTH levels. For patients with baseline iPTH < 75, ≥ 75 – < 150, ≥ 150 – ≤ 300, > 300 – ≤ 600, and > 600 pg/mL, mean monthly costs (95% CI) were estimated to be €52.68 (50.30, 55.34), €56.30 (53.78, 58.50), €62.96 (60.74, 65.07), €83.86 (81.45, 86.11), and €111.53 (108.54, 114.64), respectively. Trends were also consistent for each of the individual countries except Turkey. In Turkey the trend was reversed, with lower monthly mean costs occurring with increasing baseline iPTH levels. This could reflect the fact that in Turkey patients with elevated baseline iPTH levels and high costs were observed at the start of the follow-up period where the censoring rate was lower. Consequently, the probabilities of not being censored assigned to these costs were higher than for the costs observed in the other iPTH levels, resulting in a different trend than observed in the main analysis. Nevertheless, the two methods aim to address different aspects of the cost data.

## Discussion

The major finding of this study in patients in Western and Eastern Europe on HD is that direct medical costs were associated with high levels of iPTH and phosphate above the K/DOQI recommended range for patients with ESRD. Healthcare costs were higher with elevated phosphate and (to a lesser degree) total calcium even after adjustment for iPTH level and numerous other potentially confounding variables. Furthermore, medical costs were lower among patients with baseline phosphate levels below the K/DOQI target range.

This study assessed SHPT parameters in relation to the target ranges for patients in CKD Stage 5 recommended in the K/DOQI guidelines [5] rather than those from the more recent KDIGO guidelines [4] that were developed after the study period. The KDIGO guidelines did not prescribe numeric target ranges for these parameters, instead suggesting maintaining iPTH, total calcium, and phosphate in relation to assay reference ranges [26]. For iPTH, the range suggested in the KDIGO guidelines for CKD Stage 5 patients on dialysis corresponds to approximately 130–600 pg/mL, taking into account the different iPTH assays in use commercially [26], and therefore our findings of increased costs in patients with iPTH > 600 pg/mL would be expected to apply in relation to the KDIGO target range.

As noted above, studies in CKD patients in the US by Schumock et al. [17] and Khan et al. [18] have previously shown that SHPT can substantially increase healthcare costs. Results of these US studies are not directly comparable to ours due to patients’ different CKD stages at baseline and the focus on patients with diabetes by Schumock et al. [17] and on those with CVD by Khan et al. [18]. Nevertheless, as in the present analysis, these US studies found that elevated iPTH is associated with higher healthcare costs for CKD patients. Conversely, Smith et al. [27] found no significant association between iPTH level and healthcare costs. This contradictory finding may be due to Smith et al. [27] assigning zero cost after death, since patients who died were still included in 1-year cost calculations and mortality rate was significantly elevated in patients with higher baseline iPTH. Considerable uncertainty in the cost estimates of Smith et al. [27] should also be noted, potentially attributable to the inclusion of patients in a range of different CKD stages, primarily pre-ESRD. In contrast, the EU-FME source population we investigated had more severe disease and may represent a more clinically homogeneous group, which could help to reduce factors obscuring an influence of iPTH level on costs.

An analysis of the potential effect of SHPT treatment on rates of CVD-, fracture-, and PTX-related hospitalisation was outside the scope of the present study. This topic was previously addressed in a pooled analysis of data from 1184 subjects participating in four randomised, double-blind clinical trials of the calcimimetic SHPT medication cinacalcet, which showed a 54% reduction in the risk of fracture, a 39% reduction in the risk of CVD-related hospitalisation, and a 93% reduction in the risk of PTX for patients receiving cinacalcet compared with those receiving placebo [28].

The present study has several strengths, including use of a large, comprehensive, and validated medical database [19], which has been used in other analyses of outcomes among patients with ESRD [20,22,29]. Nevertheless, this study has a number of limitations. Generalisability of our results may be limited because the patient population represents a select group undergoing treatment at private HD facilities from a single network of providers (i.e., EU-FME). Analyses were restricted by the information available in the database; for example, costs of vitamin D use may be underestimated due to no data being collected on intravenous vitamin D use. A lower-than-expected rate of hospitalisation was observed, which could be due to missing registration in the database. The low number of hospitalisations could also be due to the relatively short person-time at risk: a mean of 13 months. A longer observational period would probably have shown more events. Finally, as with any observational study, the associations reported here are correlative only. For example, the observed association between increased medical cost and levels of iPTH and phosphate outside the K/DOQI target ranges could be driven by the effects of more advanced disease on both costs and levels of SHPT parameters, rather than by a direct effect of iPTH and phosphate levels on medical costs.

## Conclusions

This analysis indicates that elevated levels of iPTH, phosphate, and (to a lesser extent) total calcium in patients with ESRD are associated with a greater intensity of healthcare resource utilisation and higher direct medical costs in comparison with patients whose levels are within the K/DOQI target ranges. These European results support the potential association between SHPT parameters and the economic burden of SHPT in patients with CKD, as reported for patients in the US [15,18].

## Competing interests

Amgen (Europe) GmbH, the manufacturer of the calcimimetic anti-parathyroid agent cinacalcet HCl, provided financing for the publication of this article. SC, CM, and VB are employees of Amgen, and hold stocks and shares in Amgen. IG is a contractor for Amgen. LP and DM have served as consultants to Amgen, and received funding from Amgen for their participation in this study.

## Authors’ contributions

SC conceived of the study, and participated in its design and coordination. CM participated in the study design, data management, and statistical analysis. IG participated in study design, data acquisition, data management, and statistical analysis. VB participated in the study design. LP and DM participated in the study design, data acquisition, and data management. All authors contributed to interpretation of the data, helped to draft the manuscript, and read and approved the final version.

## Pre-publication history

The pre-publication history for this paper can be accessed here:

http://www.biomedcentral.com/1471-2369/13/140/prepub

## Supplementary Material

Additional file 1 **Supplementary Table S1.** ICD-10 codes used to identify hospitalisations related to cardiovascular disease, fractures, and parathyroidectomy. Description: List of ICD-10 codes used to identify SHPT-related hospitalisations in the EuCliD database. Click here for file

Additional file 2 **Supplementary Table S2.** List of medications included in estimation of healthcare resource utilisation related to secondary hyperparathyroidism, cardiovascular disease, and diabetes. Description: List of medications retrieved from the EuCliD database that were considered to be related to SHPT, CVD, and diabetes. Click here for file

Additional file 3 **Supplementary Table S3.** Patient demographics and baseline characteristics by completer status, all countries Click here for file

Additional file 4 **Supplementary Table S4.** Subcategories of healthcare costs per month by baseline iPTH, Hungary, Italy, Portugal, Spain, and Turkey. Click here for file

Additional file 5 **Supplementary Table S5.** Subcategories of healthcare costs per month by baseline total calcium, Hungary, Italy, Portugal, Spain, and Turkey. Click here for file

Additional file 6 **Supplementary Table S6.** Subcategories of healthcare costs per month by baseline phosphate, Hungary, Italy, Portugal, Spain, and Turkey. Click here for file
