# Supplementary material for: Impact of mineral and bone disorder on healthcare resource use and associated costs in the European Fresenius medical care dialysis population: a retrospective cohort study
Source: BMC Nephrol. 2012 Oct 29;13:140. doi: 10.1186/1471-2369-13-140 (PMC3504570; doi:10.1186/1471-2369-13-140)
Supplement: Additional file 1 — Supplementary Table S1. ICD-10 codes used to identify hospitalisations related to cardiovascular disease, fractures, and parathyroidectomy. Description: List of ICD-10 codes used to identify SHPT-related hospitalisations in the EuCliD database. [file 1471-2369-13-140-S1.pdf]

Supplementary Table S1. ICD-10 codes used to identify hospitalisations related to cardiovascular disease, fractures, and parathyroidectomy.

**Cardiovascular disease :**	
**Diseases of the circulatory system (I00-I99)**	
**I74**	**Arterial embolism and thrombosis**
I74.0	Embolism and thrombosis of abdominal aorta
I74.1	Embolism and thrombosis of other and unspecified parts of aorta
I74.2	Embolism and thrombosis of arteries of upper extremities
I74.3	Embolism and thrombosis of arteries of lower extremities
I74.4	Embolism and thrombosis of arteries of extremities, unspecified
I74.5	Embolism and thrombosis of iliac artery
I74.8	Embolism and thrombosis of other arteries
I74.9	Embolism and thrombosis of unspecified artery
**Hypertensive diseases (I10-I15)**	
**I10**	Essential (primary) hypertension
**I11**	**Hypertensive heart disease**
I11.0	Hypertensive heart disease with (congestive) heart failure
I11.9	Hypertensive heart disease without (congestive) heart failure
**I12**	**Hypertensive renal disease**
I12.0	Hypertensive renal disease with renal failure
I12.9	Hypertensive renal disease without renal failure
**I13**	**Hypertensive heart and renal disease**
I13.0	Hypertensive heart and renal disease with (congestive) heart failure
I13.1	Hypertensive heart and renal disease with renal failure
I13.2	Hypertensive heart and renal disease with both (congestive) heart failure and renal failure
I13.9	Hypertensive heart and renal disease, unspecified
**I15**	**Secondary hypertension**
I15.0	Renovascular hypertension
I15.1	Hypertension secondary to other renal disorders
I15.2	Hypertension secondary to endocrine disorders
I15.8	Other secondary hypertension
I15.9	Secondary hypertension, unspecified
**Ischaemic heart diseases (I20-I25)**	
**I20**	**Angina pectoris**
I20.0	Unstable angina
I20.1	Angina pectoris with documented spasm
I20.8	Other forms of angina pectoris
I20.9	Angina pectoris, unspecified
**I21**	**Acute myocardial infarction**
I21.0	Acute transmural myocardial infarction of anterior wall
I21.1	Acute transmural myocardial infarction of inferior wall
I21.2	Acute transmural myocardial infarction of other sites
I21.3	Acute transmural myocardial infarction of unspecified site
I21.4	Acute subendocardial myocardial infarction
I21.9	Acute myocardial infarction, unspecified
**I22**	**Subsequent myocardial infarction**
I22.0	Subsequent myocardial infarction of anterior wall
I22.1	Subsequent myocardial infarction of inferior wall
I22.8	Subsequent myocardial infarction of other sites
I22.9	Subsequent myocardial infarction of unspecified site

**I23**	**Certain current complications following acute myocardial infarction**
I23.0	Haemopericardium as current complication following acute myocardial infarction
I23.1	Atrial septal defect as current complication following acute myocardial infarction
I23.2	Ventricular septal defect as current complication following acute myocardial infarction
I23.3	Rupture of cardiac wall without haemopericardium as current complication following acute myocardial infarction
I23.4	Rupture of chordae tendineae as current complication following acute myocardial infarction
I23.5	Rupture of papillary muscle as current complication following acute myocardial infarction
I23.6	Thrombosis of atrium, auricular appendage, and ventricle as current complications following acute myocardial infarction
I23.8	Other current complications following acute myocardial infarction
**I24**	**Other acute ischaemic heart diseases**
I24.0	Coronary thrombosis not resulting in myocardial infarction
I24.1	Dressler's syndrome
I24.8	Other forms of acute ischaemic heart disease
I24.9	Acute ischaemic heart disease, unspecified
**I25**	**Chronic ischaemic heart disease**
I25.0	Atherosclerotic cardiovascular disease, so described
I25.1	Atherosclerotic heart disease
I25.2	Old myocardial infarction
I25.3	Aneurysm of heart
I25.4	Coronary artery aneurysm
I25.5	Ischaemic cardiomyopathy
I25.6	Silent myocardial ischaemia
I25.8	Other forms of chronic ischaemic heart disease
I25.9	Chronic ischaemic heart disease, unspecified
**Cerebrovascular diseases (I60-I69)**	
**I60**	**Subarachnoid haemorrhage**
I60.0	Subarachnoid haemorrhage from carotid siphon and bifurcation
I60.1	Subarachnoid haemorrhage from middle cerebral artery
I60.2	Subarachnoid haemorrhage from anterior communicating artery
I60.3	Subarachnoid haemorrhage from posterior communicating artery
I60.4	Subarachnoid haemorrhage from basilar artery
I60.5	Subarachnoid haemorrhage from vertebral artery
I60.6	Subarachnoid haemorrhage from other intracranial arteries
I60.7	Subarachnoid haemorrhage from intracranial artery, unspecified
I60.8	Other subarachnoid haemorrhage
I60.9	Subarachnoid haemorrhage, unspecified
**I61**	**Intracerebral haemorrhage**
I61.0	Intracerebral haemorrhage in hemisphere, subcortical
I61.1	Intracerebral haemorrhage in hemisphere, cortical
I61.2	Intracerebral haemorrhage in hemisphere, unspecified
I61.3	Intracerebral haemorrhage in brain stem
I61.4	Intracerebral haemorrhage in cerebellum
I61.5	Intracerebral haemorrhage, intraventricular
I61.6	Intracerebral haemorrhage, multiple localized
I61.8	Other intracerebral haemorrhage
I61.9	Intracerebral haemorrhage, unspecified
**I62**	**Other nontraumatic intracranial haemorrhage**
I62.0	Subdural haemorrhage (acute)(nontraumatic)
I62.1	Nontraumatic extradural haemorrhage
I62.9	Intracranial haemorrhage (nontraumatic), unspecified
**I63**	**Cerebral infarction**
I63.0	Cerebral infarction due to thrombosis of precerebral arteries
I63.1	Cerebral infarction due to embolism of precerebral arteries

I63.2	Cerebral infarction due to unspecified occlusion or stenosis of precerebral arteries
I63.3	Cerebral infarction due to thrombosis of cerebral arteries
I63.4	Cerebral infarction due to embolism of cerebral arteries
I63.5	Cerebral infarction due to unspecified occlusion or stenosis of cerebral arteries
I63.6	Cerebral infarction due to cerebral venous thrombosis, nonpyogenic
I63.8	Other cerebral infarction
I63.9	Cerebral infarction, unspecified
**I64**	**Stroke, not specified as haemorrhage or infarction**
**I65**	**Occlusion and stenosis of precerebral arteries, not resulting in cerebral infarction**
I65.0	Occlusion and stenosis of vertebral artery
I65.1	Occlusion and stenosis of basilar artery
I65.2	Occlusion and stenosis of carotid artery
I65.3	Occlusion and stenosis of multiple and bilateral precerebral arteries
I65.8	Occlusion and stenosis of other precerebral artery
I65.9	Occlusion and stenosis of unspecified precerebral artery
**I66**	**Occlusion and stenosis of cerebral arteries, not resulting in cerebral infarction**
I66.0	Occlusion and stenosis of middle cerebral artery
I66.1	Occlusion and stenosis of anterior cerebral artery
I66.2	Occlusion and stenosis of posterior cerebral artery
I66.3	Occlusion and stenosis of cerebellar arteries
I66.4	Occlusion and stenosis of multiple and bilateral cerebral arteries
I66.8	Occlusion and stenosis of other cerebral artery
I66.9	Occlusion and stenosis of unspecified cerebral artery
**I67**	**Other cerebrovascular diseases**
I67.0	Dissection of cerebral arteries, nonruptured
I67.1	Cerebral aneurysm, nonruptured
I67.2	Cerebral atherosclerosis
I67.3	Progressive vascular leukoencephalopathy
I67.4	Hypertensive encephalopathy
I67.5	Moyamoya disease
I67.6	Nonpyogenic thrombosis of intracranial venous system
I67.7	Cerebral arteritis, not elsewhere classified
I67.8	Other specified cerebrovascular diseases
I67.9	Cerebrovascular disease, unspecified
**I68**	**Cerebrovascular disorders in diseases classified elsewhere**
I68.0	Cerebral amyloid angiopathy (E85.-+)
I68.1	Cerebral arteritis in infectious and parasitic diseases classified elsewhere
I68.2	Cerebral arteritis in other diseases classified elsewhere
I68.8	Other cerebrovascular disorders in diseases classified elsewhere
**I69**	**Sequelae of cerebrovascular disease**
I69.0	Sequelae of subarachnoid haemorrhage
I69.1	Sequelae of intracerebral haemorrhage
I69.2	Sequelae of other nontraumatic intracranial haemorrhage
I69.3	Sequelae of cerebral infarction
I69.4	Sequelae of stroke, not specified as haemorrhage or infarction
I69.8	Sequelae of other and unspecified cerebrovascular diseases
**Diseases of the nervous system (G00-G99)**	
**G45**	**Transient cerebral ischaemic attacks and related syndromes**
G45.0	Vertebro-basilar artery syndrome
G45.1	Carotid artery syndrome (hemispheric)
G45.2	Multiple and bilateral precerebral artery syndromes
G45.3	Amaurosis fugax
G45.4	Transient global amnesia
G45.8	Other transient cerebral ischaemic attacks and related syndromes
G45.9	Transient cerebral ischaemic attack, unspecified

Fractures:		
	**S12**	**Fracture of neck**
	S12.0	Fracture of first cervical vertebra
	S12.1	Fracture of second cervical vertebra
	S12.2	Fracture of other specified cervical vertebra
	S12.7	Multiple fractures of cervical spine
	S12.9	Fracture of neck, part unspecified
	**S22**	**Fracture of rib(s), sternum and thoracic spine**
	S22.0	Fracture of thoracic vertebra
	S22.1	Multiple fractures of thoracic spine
	S22.2	Fracture of sternum
	S22.3	Fracture of rib(s), sternum and thoracic spine
	S22.4	Multiple fractures of ribs
	S22.5	Flail chest
	S22.8	Fracture of other parts of bony thorax
	S22.9	Fracture of bony thorax, part unspecified
	**S32**	**Fracture of lumbar spine and pelvis**
	S32.0	Fracture of lumbar vertebra
	S32.1	Fracture of sacrum
	S32.2	Fracture of coccyx
	S32.3	Fracture of ilium
	S32.4	Fracture of acetabulum
	S32.5	Fracture of pubis
	S32.7	Multiple fractures of lumbar spine and pelvis
	S32.8	Fracture of other and unspecified parts of lumbar spine and pelvis
	**S42**	**Fracture of shoulder and upper arm**
	S42.0	Fracture of clavicle
	S42.1	Fracture of scapula
	S42.2	Fracture of upper end of humerus
	S42.3	Fracture of shaft of humerus
	S42.4	Fracture of lower end of humerus
	S42.7	Multiple fractures of clavicle, scapula and humerus
	S42.8	Fracture of other parts of shoulder and upper arm
	S42.9	Fracture of shoulder girdle, part unspecified
	**S52**	**Fracture of forearm**
	S52.0	Fracture of upper end of ulna
	S52.1	Fracture of upper end of radius
	S52.3	Fracture of shaft of ulna
	S52.4	Fracture of shaft of radius
	S52.5	Fracture of shaft of both ulna and radius
	S52.6	Fracture of lower end of both ulna and radius
	S52.7	Multiple fractures of forearm
	S52.8	Fracture of other parts of forearm
	S52.9	Fracture of forearm, part unspecified
	**S62**	**Fracture at wrist and hand level**
	S62.0	Fracture of navicular [scaphoid] bone of hand
	S62.1	Fracture of other carpal bone(s)
	S62.3	Fracture of other metacarpal bone
	S62.4	Multiple fractures of metacarpal bones
	S62.5	Fracture of thumb
	S62.6	Fracture of other finger
	S62.7	Multiple fracture of fingers
	S62.8	Fracture of other and unspecified parts of wrist and hand
	**S72**	**Fracture of femur**
	S72.0	Fracture of neck of femur
	S72.1	Pertrochanteric fracture

S72.2	Subtrochanteric fracture
S72.3	Fracture of the shaft of femur
S72.4	Fracture of lower end of femur
S72.7	Multiple fractures of femur
S72.8	Fractures of other parts of femur
S72.9	Fracture of femur, part unspecified
**S82**	**Fracture of lower leg, including ankle**
S82.1	Fracture of upper end of tibia
S82.2	Fracture of shaft of tibia
S82.3	Fracture of lower end of tibia
S82.4	Fracture of fibula alone
S82.5	Fracture of medial malleolus
S82.6	Fracture of lateral malleolus
S82.7	Multiple fractures of lower leg
S82.8	Fractures of other parts of lower leg
S82.9	Fracture of lower leg, part unspecified
**S92**	**Fracture of foot, except ankle**
S92.0	Fracture of calcaneus
S92.1	Fracture of talus
S92.2	Fracture of other tarsal bone(s)
S92.3	Fracture of metatarsal bone
S92.4	Fracture of great toe
S92.5	Fracture of other toe
S92.7	Multiple fractures of foot
S92.9	Fracture of foot, unspecified
**T02**	**Fractures involving multiple body regions**
T02.1	Fractures involving thorax with lower back and pelvis
T02.2	Fractures involving multiple regions of one upper limb
T02.3	Fractures involving multiple regions of one lower limb
T02.4	Fractures involving multiple regions of both upper limbs
T02.5	Fractures involving multiple regions of both lower limbs
T02.6	Fractures involving multiple regions of upper limb(s) with lower limb(s)
T02.7	Fractures involving thorax with lower back and pelvis with limb(s)
T02.8	Fractures involving other combinations of body regions
T02.9	Multiple fractures, unspecified
**T08**	**Fracture of spine, level unspecified**
**T10**	**Fracture of upper limb, level unspecified**
**T12**	**Fracture of lower limb, level unspecified**
**T14**	**Injury of unspecified body region**
T14.2	Fracture of unspecified body region
**T91**	**Sequelae of injuries of neck and trunk**
T91.1	Sequelae of fracture of spine
T91.2	Sequelae of other fracture of thorax and pelvis
**T92**	**Sequelae of injuries of upper limb**
T92.1	Sequelea of fracture of arm
T92.2	Sequelea of fracture at wrist and hand level
**T93**	**Sequelae of injuries of lower limb**
T93.1	Sequelae of fracture of femur
T93.2	Sequelae of other fractures of lower limb
**M80**	**Osteoporosis with pathological fracture**
M80.5	Idiopathic osteoporosis with pathological fracture
M80.8	Other osteoporosis with pathological fracture
M80.9	Unspecified osteoporosis with pathological fracture
**M84**	**Disorders of continuity of bone**
M84.4	Pathological fracture, not elsewhere classified

**Parathyroidectomy:**	
**E21**	**Hyperparathyroidism and other disorders of parathyroid gland**
E21.1	Secondary hyperparathyroidism, not elsewhere classified
E21.2	Other hyperparathyroidism
E21.3	Hyperparathyroidism, unspecified

Source: **International Statistical Classification of Diseases and Related Health Problems 10th Revision** [http://apps.who.int/classifications/icd10/browse/2010/en]
