# Supplementary material for: Impact of mineral and bone disorder on healthcare resource use and associated costs in the European Fresenius medical care dialysis population: a retrospective cohort study
Source: BMC Nephrol. 2012 Oct 29;13:140. doi: 10.1186/1471-2369-13-140 (PMC3504570; doi:10.1186/1471-2369-13-140)
Supplement: Additional file 2 — Supplementary Table S2. List of medications included in estimation of healthcare resource utilisation related to secondary hyperparathyroidism, cardiovascular disease, and diabetes. Description: List of medications retrieved from the EuCliD database that were considered to be related to SHPT, CVD, and diabetes. [file 1471-2369-13-140-S2.pdf]

Supplementary Table S2. List of medications included in estimation of healthcare resource utilisation related to secondary hyperparathyroidism, cardiovascular disease, and diabetes.

Drug Class	Generic Name
**Secondary hyperparathyroidism**	
Phosphate binder	Aluminium hydroxide
	Aluminium phosphate
	Calcium acetate
	Calcium acetate/magnesium carbonate
	Calcium carbonate
	Calcium glubionate
	Calcium gluconate
	Calcium phosphate
	Calcium (different salts in combination)
	Lanthanum carbonate
	Polystyrene sulfonate
	Sevelamer
Oral vitamin D sterol	Alfacalcidol
	Calcifediol
	Calcitriol
	Colecalciferol
	Doxercalciferol
	Ergocalciferol
	Paricalcitol
Calcimimetic	Cinacalcet
**Cardiovascular disease**	
Anti-aggregant	
Cyclooxygenase inhibitor	Acetylsalicylic acid
	Acetylsalicylic acid, combinations with psycholeptics
	Carbasalate calcium
ADP-receptor inhibitor	Clopidogrel
	Prasugrel
	Ticlopidine
Phosphodiesterase inhibitor	Cilostazol
Glycoprotein IIB/IIIA inhibitor (IV use only)	Abciximab
	Eptifibatide
	Fondaparinux
	Tirofiban
	Defibrotide
Adenosine reuptake inhibitor	Dipyridamole
Statin	Atorvastatin
	Fluvastatin
	Lovastatin
	Mevastatin
	Pitavastatin
	Pravastatin
	Rosuvastatin
	Simvastatin
	Atorvastatin + amlodipine besylate
	Lovastatin + niacin extended-release
	Simvastatin + ezetimibe
	Simvastatin + niacin extended-release
Peripheral vasodilator	Buflomedil
	Ifenprodil
	Naftidrofuryl

Drug Class	Generic Name
	Vincamine
Fibrate	Bezafibrate
	Ciprofibrate
	Clofibrate
	Gemfibrozil
	Fenofibrate
Antihypertensive	
Diuretic	
Loop diuretic	Bumetanide
	Ethacrynic acid
	Furosemide
	Piretanide
	Torasemide
Thiazide diuretic	Bendroflumethiazide
	Epitizide
	Hydrochlorothiazide and chlorothiazide
Thiazide-like diuretic	Chlortalidone
	Indapamide
	Metolazone
	Xipamide
Potassium-sparing diuretic	Amiloride
	Potassium canrenoate
	Spironolactone
	Triamterene
Adrenergic receptor antagonist	
Beta blocker	Acebutolol
	Atenolol
	Betaxolol
	Bisoprolol
	Carteolol
	Celiprolol
	Metoprolol
	Nadolol
	Nebivolol
	Oxprenolol
	Pindolol
	Propranolol
	Solatol
	Tertatolol
	Timolol
Alpha blocker	Doxazosin
	Indoramin
	Phenoxybenzamine
	Phentolamine
	Prazosin
	Terazosin
	Tolazoline
	Uradipil
Mixed alpha + beta blocker	Bucindolol
	Carvedilol
	Labetalol
Adrenergic receptor agonist	
Alpha-2 agonist	Clonidine
	Guanfacine

Drug Class	Generic Name
	Methyldopa
	Moxonidine
	Rilmenidine
Calcium channel blocker	
Dihydropyridine	Amlodipine
	Cyclandelate
	Felodipine
	Isradipine
	Lacidipine
	Lercanidipine
	Manidipine
	Nicardipine
	Nifedipine
	Nimodipine
	Nitrendipine
Non-dihydropyridine	Diltiazem
	Verapamil
ACE inhibitor	Benazepril
	Captopril
	Cilazapril
	Enalapril
	Fosinopril
	Imadapril
	Lisinopril
	Moexipril
	Perindopril
	Quinapril
	Ramipril
	Trandolapril
	Zofenopril
Angiotensin II receptor antagonist	Candesartan
	Eprosartan
	Irbesartan
	Losartan
	Olmesartan medoxomil
	Telmisartan
	Valsartan
Aldosterone antagonist	Eplerenone
	Spironolactone
Oral anticoagulant	Acenocoumarol
	Phenindione
	Tioclomarol
	Warfarin
**Diabetes**	
Insulin	
Regular insulin	Regular
Rapid-acting	Insulin (human)
	Insulin (pork)
	Insulin aspart
	Insulin glulisine
	Insulin lispro
Intermediate-acting	Insulin (pork)
	NPH
	Combinations

Drug Class	Generic Name
Long-acting	Insulin (beef)
	Insulin (pork)
	Insulin detemir
	Insulin glargine
	Combinations
Mixtures	50% lispro protamine, 50% insulin lispro
	70% aspart protamine, 30% aspart
	70% NPH, 30% regular
	70% NPH, 30% regular
	75% lispro protamine, 25% insulin lispro
	Insulin (pork)
Sulfonylureas	
First-generation agents	Acetohexamide
	Carbutamide
	Chlorpropamide
	Tolazamide
	Tolbutamide
Second-generation agents	Glibonuride
	Gliclazide
	Glimepiride
	Glipizide
	Glyburide
Meglitinides	Nateglinide
	Repaglinide
Biguanides	Metformin
Thiazolidinediones	Pioglitazone
	Rosiglitazone
Alpha-glucosidase inhibitors	Acarbose
	Miglitol
GLP analogues	Benfluorex
	Exenatide
	Liraglutide
DPP-4 inhibitors	Sitagliptin
	Vildagliptin

Abbreviations: ACE, angiotensin-converting enzyme; ADP, adenosine diphosphate; DPP, dipeptidyl peptidase; GLP, glucagon-like peptide; IV, intravenous; NPH, neutral protamine Hagedorn
