# Supplementary material for: Impact of mineral and bone disorder on healthcare resource use and associated costs in the European Fresenius medical care dialysis population: a retrospective cohort study
Source: BMC Nephrol. 2012 Oct 29;13:140. doi: 10.1186/1471-2369-13-140 (PMC3504570; doi:10.1186/1471-2369-13-140)
Supplement: Additional file 3 — Supplementary Table S3. Patient demographics and baseline characteristics by completer status, all countries [file 1471-2369-13-140-S3.pdf]

Supplementary Table S3. Patient demographics and baseline characteristics by completer status, all countries.

	Completed Follow-up Period (N = 4873)	Did Not Complete Follow-up Period (N = 1496)	Total (N = 6369)
Males, n (%)	2753 (56)	877 (59)	3630 (57)
Age, years			
n	4873	1496	6369
Mean	62.46	64.82	63.02
SD	14.65	14.72	14.70
Median	65.00	67.00	65.00
Q1, Q3	53.00, 74.00	56.00, 76.00	53.00, 74.00
Min, Max	18.0, 101.0	18.0, 97.0	18.0, 101.0
History, n (%)			
Diabetes	1131 (23)	450 (30)	1581 (25)
CVD	3722 (76)	1242 (83)	4964 (78)
Cancer	273 (6)	111 (7)	384 (6)
CKD aetiology, n (%)			
Hypertension/vascular	685 (14)	201 (13)	886 (14)
Glomerulonephritis	810 (17)	258 (17)	1068 (17)
Diabetes	653 (13)	238 (16)	891 (14)
Tubulo-interstitial	684 (14)	192 (13)	876 (14)
Polycystic kidney disease	302 (6)	81 (5)	383 (6)
Miscellaneous	199 (4)	51 (3)	250 (4)
Unknown	1143 (23)	353 (24)	1496 (23)
Missing	397 (8)	122 (8)	519 (8)
iPTH, pg/mL			
n	4873	1496	6369
Mean	299.46	312.21	302.45
SD	338.97	369.09	346.29
Median	192.10	193.83	192.90
Q1, Q3	96.05, 369.50	88.74, 379.50	94.00, 372.00
Min, Max	1.7, 2985.7	1.0, 2824.8	1.0, 2985.7
Total calcium, mmol/L			
n	4776	1424	6200
Mean	2.28	2.27	2.28
SD	0.20	0.20	0.20
Median	2.26	2.26	2.26
Q1, Q3	2.16, 2.38	2.15, 2.38	2.15, 2.38
Min, Max	1.3, 3.7	1.6, 3.6	1.3, 3.7
Phosphate, mmol/L			
n	4706	1415	6121
Mean	1.53	1.51	1.53
SD	0.39	0.42	0.40
Median	1.50	1.47	1.49
Q1, Q3	1.26, 1.77	1.22, 1.78	1.25, 1.77
Min, Max	0.5, 2.9	0.5, 2.9	0.5, 2.9
CRP, mg/L			
n	3433	1063	4496
Mean	11.77	14.43	12.40
SD	15.73	18.17	16.38
Median	6.67	8.00	7.00
Q1, Q3	3.00, 14.00	3.53, 18.40	3.10, 14.88
Min, Max	0.1, 171.0	0.1, 170.0	0.1, 171.0

	Completed Follow-up Period (N = 4873)	Did Not Complete Follow-up Period (N = 1496)	Total (N = 6369)
Serum albumin, g/dL			
n	4449	1304	5753
Mean	3.94	3.84	3.92
SD	0.48	0.56	0.50
Median	3.95	3.87	3.92
Q1, Q3	3.69, 4.19	3.56, 4.13	3.65, 4.17
Min, Max	2.1, 6.6	1.6, 7.0	1.6, 7.0
Haemoglobin, g/dL			
n	4840	1468	6308
Mean	11.54	11.49	11.53
SD	1.41	1.49	1.43
Median	11.56	11.50	11.53
Q1, Q3	10.63, 12.46	10.55, 12.50	10.60, 12.47
Min, Max	5.5, 16.8	7.2, 15.8	5.5, 16.8
Ferritin, µg/L			
n	4615	1408	6023
Mean	527.27	537.86	529.74
SD	504.85	478.07	498.70
Median	400.00	409.83	402.27
Q1, Q3	204.00, 695.00	220.00, 729.00	207.20, 702.50
Min, Max	2.3, 8241.0	7.0, 4208.4	2.3, 8241.0
Cholesterol, mmol/L			
n	3944	1158	5102
Mean	4.43	4.33	4.40
SD	1.10	1.13	1.11
Median	4.32	4.20	4.30
Q1, Q3	3.68, 5.06	3.52, 5.00	3.63, 5.05
Min, Max	1.2, 12.0	1.3, 8.7	1.2, 12.0
Blood Leucocytes, no./mm3			
n	3978	1309	5287
Mean	6879.2	7098.4	6933.5
SD	2135.4	2259.7	2168.7
Median	6725.8	6900.0	6766.7
Q1, Q3	5550.0, 8033.3	5600.0, 8320.0	5566.7, 8116.7
Min, Max	122, 24600	300, 20420	122, 24600
Dialysis vintage*, months			
n	4873	1496	6369
Mean	45.389	45.997	45.532
SD	55.297	58.380	56.032
Median	26.908	27.663	27.203
Q1, Q3	5.355, 62.357	7.901, 61.569	6.045, 62.193
Min, Max	0.03, 476.65	0.03, 834.79	0.03, 834.79

CKD, chronic kidney disease; CRP, C-reactive protein; CVD, cardiovascular disease; iPTH, intact parathyroid hormone; SHPT, secondary hyperparathyroidism

*Calculated from initiation of dialysis to the start of the 3-month exposure period.
