# Supplementary material for: Impact of mineral and bone disorder on healthcare resource use and associated costs in the European Fresenius medical care dialysis population: a retrospective cohort study
Source: BMC Nephrol. 2012 Oct 29;13:140. doi: 10.1186/1471-2369-13-140 (PMC3504570; doi:10.1186/1471-2369-13-140)
Supplement: Additional file 4 — Supplementary Table S4. Subcategories of healthcare costs per month by baseline iPTH, Hungary, Italy, Portugal, Spain, and Turkey. [file 1471-2369-13-140-S4.pdf]

Supplementary Table S4. Subcategories of healthcare costs per month by baseline iPTH, Hungary, Italy, Portugal, Spain, and Turkey.

	Baseline iPTH*, pg/mL						
	< 75	≥ 75 – < 150	≥ 150 – ≤ 300	> 300 – ≤ 600	> 600	> 800	Total
N patients	1054	1145	1394	1140	653	405	5386
Cost of CVD-related hospitalisations per month (including patients with zero costs), 2006 €							
Mean	17.63	17.79	19.08	14.87	14.06	15.66	17.02
SD	178.31	287.65	249.22	133.97	109.49	128.88	212.40
Median	0.00	0.00	0.00	0.00	0.00	0.00	0.00
Q1, Q3	0.00, 0.00	0.00, 0.00	0.00, 0.00	0.00, 0.00	0.00, 0.00	0.00, 0.00	0.00, 0.00
Min, Max	0.00, 3879.20	0.00, 9533.54	0.00, 7501.80	0.00, 3790.69	0.00, 1879.98	0.00, 1879.98	0.00, 9533.54
Patients with CVD-related hospitalisation cost per month > €0, n (%)	26 (2)	37 (3)	44 (3)	51 (4)	22 (3)	13 (3)	180 (3)
Cost of fracture-related hospitalisations per month (including patients with zero costs), 2006 €							
Mean	3.90	4.24	1.75	2.71	4.24	3.53	3.20
SD	44.95	62.95	29.81	28.63	47.48	49.14	43.75
Median	0.00	0.00	0.00	0.00	0.00	0.00	0.00
Q1, Q3	0.00, 0.00	0.00, 0.00	0.00, 0.00	0.00, 0.00	0.00, 0.00	0.00, 0.00	0.00, 0.00
Min, Max	0.00, 857.85	0.00, 1762.45	0.00, 764.75	0.00, 549.49	0.00, 941.99	0.00, 941.99	0.00, 1762.45
Patients with fracture-related hospitalisation cost per month > €0, n (%)	11 (1)	10 (1)	7 (1)	12 (1)	8 (1)	4 (1)	48 (1)
Cost of parathyroidectomy-related hospitalisations per month (including patients with zero costs), 2006 €							
Mean	1.87	1.18	0.67	0.29	7.21	10.30	1.73
SD	38.31	27.36	25.01	7.02	54.86	67.80	31.42
Median	0.00	0.00	0.00	0.00	0.00	0.00	0.00
Q1, Q3	0.00, 0.00	0.00, 0.00	0.00, 0.00	0.00, 0.00	0.00, 0.00	0.00, 0.00	0.00, 0.00
Min, Max	0.00, 1133.29	0.00, 858.30	0.00, 933.83	0.00, 189.19	0.00, 1133.29	0.00, 1133.29	0.00, 1133.29
Patients with parathyroidectomy-related hospitalisation cost per month > €0, n (%)	4 (0)	3 (0)	1 (0)	2 (0)	20 (3)	17 (4)	30 (1)

	Baseline iPTH*, pg/mL						
	< 75	≥ 75 – < 150	≥ 150 – ≤ 300	> 300 – ≤ 600	> 600	> 800	Total
Cost of CVD medications per month (including patients with zero costs), 2006 €							
Mean	13.10	14.39	15.63	16.09	14.71	13.71	14.86
SD	23.02	23.70	23.46	23.12	22.10	22.44	23.21
Median	3.51	5.34	5.52	5.51	5.32	4.77	5.00
Q1, Q3	0.00, 15.33	0.00, 18.19	0.03, 20.92	0.00, 23.07	0.03, 19.41	0.00, 16.84	0.00, 19.41
Min, Max	0.00, 217.84	0.00, 331.96	0.00, 197.79	0.00, 175.38	0.00, 211.68	0.00, 211.68	0.00, 331.96
Patients with CVD medication cost per month > €0, n (%)	722 (69)	844 (74)	1049 (75)	844 (74)	490 (75)	300 (74)	3949 (73)
Cost of SHPT medications per month (including patients with zero costs), 2006 €							
Mean	19.83	26.49	32.43	46.96	69.11	70.48	36.22
SD	35.96	44.92	54.89	67.07	96.20	100.49	61.36
Median	5.38	6.42	8.77	18.42	36.96	36.96	9.39
Q1, Q3	0.84, 19.58	1.67, 32.93	2.23, 39.38	3.23, 68.15	5.42, 83.99	5.54, 85.18	2.26, 44.95
Min, Max	0.00, 295.38	0.00, 329.59	0.00, 474.57	0.00, 461.33	0.00, 662.47	0.00, 662.47	0.00, 662.47
Patients with SHPT medication cost per month > €0, n (%)	827 (78)	942 (82)	1181 (85)	978 (86)	575 (88)	357 (88)	4503 (84)
Cost of diabetes medications per month (including patients with zero costs), 2006 €							
Mean	4.19	2.93	3.91	1.54	0.63	0.45	2.86
SD	24.09	15.02	28.79	13.79	3.31	2.36	20.47
Median	0.00	0.00	0.00	0.00	0.00	0.00	0.00
Q1, Q3	0.00, 0.00	0.00, 0.00	0.00, 0.00	0.00, 0.00	0.00, 0.00	0.00, 0.00	0.00, 0.00
Min, Max	0.00, 275.14	0.00, 199.84	0.00, 554.20	0.00, 352.22	0.00, 41.31	0.00, 30.50	0.00, 554.20
Patients with diabetes medication cost per month > €0, n (%)	157 (15)	186 (16)	195 (14)	117 (10)	50 (8)	26 (6)	705 (13)

CVD, cardiovascular disease; iPTH, intact parathyroid hormone; SHPT, secondary hyperparathyroidism
*Mean iPTH during 3-month baseline period
