# Supplementary material for: Impact of mineral and bone disorder on healthcare resource use and associated costs in the European Fresenius medical care dialysis population: a retrospective cohort study
Source: BMC Nephrol. 2012 Oct 29;13:140. doi: 10.1186/1471-2369-13-140 (PMC3504570; doi:10.1186/1471-2369-13-140)
Supplement: Additional file 6 — Supplementary Table S6. Subcategories of healthcare costs per month by baseline phosphate, Hungary, Italy, Portugal, Spain, and Turkey. [file 1471-2369-13-140-S6.pdf]

Supplementary Table S6. Subcategories of healthcare costs per month by baseline phosphate, Hungary, Italy, Portugal, Spain, and Turkey.

	Baseline phosphate*, mmol/mL				
	< 1.13	≥ 1.13 – ≤ 1.78	> 1.78	Missing	Total
N patients	827	3104	1208	247	5386
Cost of CVD-related hospitalisations per month (including patients with zero costs), 2006 €					
Mean	16.47	18.30	13.22	21.41	17.02
SD	150.00	259.92	97.81	113.06	212.40
Median	0.00	0.00	0.00	0.00	0.00
Q1, Q3	0.00, 0.00	0.00, 0.00	0.00, 0.00	0.00, 0.00	0.00, 0.00
Min, Max	0.00, 3790.69	0.00, 9533.54	0.00, 1879.98	0.00, 1142.98	0.00, 9533.54
Patients with CVD-related hospitalisation cost per month > €0, n (%)	33 (4)	91 (3)	43 (4)	13 (5)	180 (3)
Cost of fracture-related hospitalisations per month (including patients with zero costs), 2006 €					
Mean	6.06	3.02	1.63	3.63	3.20
SD	56.61	44.35	30.00	43.25	43.75
Median	0.00	0.00	0.00	0.00	0.00
Q1, Q3	0.00, 0.00	0.00, 0.00	0.00, 0.00	0.00, 0.00	0.00, 0.00
Min, Max	0.00, 857.85	0.00, 1762.45	0.00, 941.99	0.00, 623.02	0.00, 1762.45
Patients with fracture-related hospitalisation cost per month > €0, n (%)	13 (2)	26 (1)	7 (1)	2 (1)	48 (1)
Cost of parathyroidectomy-related hospitalisations per month (including patients with zero costs), 2006 €					
Mean	2.28	1.21	3.01	0.00	1.73
SD	42.76	25.18	38.98	0.00	31.42
Median	0.00	0.00	0.00	0.00	0.00
Q1, Q3	0.00, 0.00	0.00, 0.00	0.00, 0.00	0.00, 0.00	0.00, 0.00
Min, Max	0.00, 1133.29	0.00, 933.83	0.00, 1133.29	0.00, 0.00	0.00, 1133.29
Patients with parathyroidectomy-related hospitalisation cost per month > €0, n (%)	4 (0)	13 (0)	13 (1)	0 (0)	30 (1)
Cost of CVD medications per month (including patients with zero costs), 2006 €					
Mean	14.64	14.96	14.53	15.84	14.86
SD	23.96	23.20	23.42	19.42	23.21
Median	3.53	4.76	5.82	9.04	5.00
Q1, Q3	0.00, 18.61	0.00, 20.10	0.05, 17.88	0.94, 22.23	0.00, 19.41
Min, Max	0.00, 217.84	0.00, 211.68	0.00, 331.96	0.00, 104.44	0.00, 331.96
Patients with CVD medication cost per month > €0, n (%)	574 (69)	2274 (73)	908 (75)	193 (78)	3949 (73)

	Baseline phosphate*, mmol/mL				
	< 1.13	≥ 1.13 – ≤ 1.78	> 1.78	Missing	Total
Cost of SHPT medications per month (including patients with zero costs), 2006 €					
Mean	18.70	34.62	54.66	24.89	36.22
SD	40.31	57.64	78.93	40.22	61.36
Median	4.27	9.59	27.03	5.71	9.39
Q1, Q3	0.00, 11.78	2.26, 41.68	4.60, 74.10	1.51, 36.08	2.26, 44.95
Min, Max	0.00, 326.86	0.00, 502.02	0.00, 662.47	0.00, 365.41	0.00, 662.47
Patients with SHPT medication cost per month > €0, n (%)	615 (74)	2618 (84)	1059 (88)	211 (85)	4503 (84)
Cost of diabetes medications per month (including patients with zero costs), 2006 €					
Mean	3.87	3.16	1.82	0.82	2.86
SD	25.49	21.03	16.73	4.80	20.47
Median	0.00	0.00	0.00	0.00	0.00
Q1, Q3	0.00, 0.00	0.00, 0.00	0.00, 0.00	0.00, 0.00	0.00, 0.00
Min, Max	0.00, 366.86	0.00, 554.20	0.00, 450.89	0.00, 67.10	0.00, 554.20
Patients with diabetes medication cost per month > €0, n (%)	131 (16)	428 (14)	121 (10)	25 (10)	705 (13)

CVD, cardiovascular disease; SHPT, secondary hyperparathyroidism
*Mean phosphate during 3-month baseline period
